# Scattering on scattering

**DOI:** 10.1038/lsa.2017.88

**Published:** 2017-07-28

**Authors:** Jörn Bonse

**Affiliations:** 1 Fachbereich 6.4 Technologien mit Nanowerkstoffen, Bundesanstalt für Materialforschung und -prüfung (BAM), Unter den Eichen 87, Berlin D-12205, Germany

**Ultrafast experiments can reveal the spatiotemporal dynamics of nanostructure formation via scattering in background-free optical dark-field microscopy.**

Scattering of electromagnetic radiation is used in many scientific, industrial and daily applications. In time-resolved experiments using ultrafast lasers, the spatial and temporal coherence of the radiation is of crucial importance for visualizing scattering phenomena. On the macro-scale, an ultrafast video recording at 100 billion frames per second can impressively visualize even the Mach cone superluminal propagation of light in scattering media^1^; however, imaging objects at the micro-scale via ultrafast microscopy is often challenging: the fact that the coherence of the illuminating laser radiation is manifested in undesired speckle patterns can limit the spatial resolution and thus the single-shot imaging capabilities^2^. On the other hand, the ability of coherent radiation to interfere in space and time can be constructively used to reveal the ultrafast dynamics of nanostructure formation via coherent scattering/diffraction in the reciprocal space^3^.

Time-resolved microscopy techniques (in the real or the reciprocal space) offer unique capabilities of monitoring extremely fast and irreversible processes, such as ultrafast phase transitions during the laser processing of solids, including non-thermal or thermal melting, ablation and resolidification phenomena. For instance, pump-probe bright-field microscopy records snapshots of the surface reflectivity or transmittivity at different time delays after the pump pulse has hit the sample surface (see Figure 1a). The extraordinarily high temporal resolution of this technique is a result of the duration of the probe pulse, which is typically 100 fs. Starting from the initial idea of Downer *et al.*^4^, Sokolowski-Tinten and von der Linde extended the technique^5^ and added new modalities^6^. These extensions led to significant contributions to the understanding of complex laser ablation phenomena.

In a recent publication, Fang *et al.*^7^ present a dark-field variant of the original pump-probe microscopy system, extending the functionality of this technology toward the microsecond range and enabling the capture of the complete spatiotemporal evolution of femtosecond-laser-induced modifications at the surface of strongly absorbing materials such as metals. This new variant is based on optical radiation scattering and thus, provides complementary information to that provided by normal bright-field microscopy. The approach of Fang *et al.* follows the historical idea of ultramicroscopy for the visualization of sub-wavelength structures (awarded the Nobel Prize in Chemistry to R.A. Zsigmondy in 1925) and combines the advantage of zero-background high-contrast imaging of scattered light with the temporal resolution provided by modern femtosecond-laser technology. In particular, this imaging technique is sensitive to micro- and nanoscale changes of the surface topography as well as to sub- and supra-wavelength-sized particles ablating from it. The basic idea is outlined in Figure 1b. Compared to bright-field pump-probe microscopy illustrated in Figure 1a, in the dark-field version, the illuminating probe beam is incident under a small angle to the surface. However, the angle is large enough to prevent the specular reflected beam from being collected by the imaging optics oriented normal to the sample surface. In both microscopy variants, the pump beam excites a surface region within the field of view of the microscope and triggers a complex chain of processes following the laser pulse absorption by the electronic system of the sample material. This may involve melt flows driven by capillary forces, the ablational defragmentation of a near surface layer into atoms and ions, or scattering by micro- and nanoparticles, mass density changes or other relaxation processes. Subsequently, the delayed probe beam illuminates the transient scenario and redirects light via scattering towards a synchronized camera-based imaging system. This dark-field illumination scheme (Figure 1b) elegantly suppresses the contribution of specular reflection to the time-resolved frames captured by the camera. The specular reflection is strongly affected by the electronic configuration of the laser-irradiated material, while the scattered light is responsive to the topography of the material. Hence, the dark-field imaging allows the separation of both contributions, which are superimposed in bright-field imaging.

Exploiting the high sensitivity to topographical changes and ablated particles, Fang *et al.* reported the onset of optical scattering at a wavelength of 400 nm (probe) as an indication of material displacement ~100 ps after the impact of a single 65-fs pump pulse at a wavelength of 800 nm onto a polished zinc surface^7^. Tracking the complete sequence of complex follow-up processes, the final surface state was reached after a few hundred nanoseconds. The new paper of Fang *et al.*^7^ makes an important step by transferring another variant of ‘classical microscopy’ into an ultrafast pump-probe microscopy scheme. To date, this was realized as time-resolved bright-field microscopy in reflection^5^ or transmission^8^, as ultrafast imaging interferometry^6^ or as Nomarsky^9^ and phase-contrast microscope versions^2^.

The new dark-field method^7^ could be extended by utilizing tunable probe wavelengths to provide indirect information on the size of ablated particles when testing against the models based on Rayleigh or Mie scattering. Furthermore, a random laser^2^ may be employed for reducing the coherence of the probe beam radiation, improving the spatial resolution via a reduction of speckles. It can be expected that the approach of Fang *et al.* will trigger further research, leading to new results on the dynamics of ablated particles and on the formation of transient surface structures, thus contributing to a better understanding of laser ablation phenomena.

## Figures and Tables

**Figure 1:**
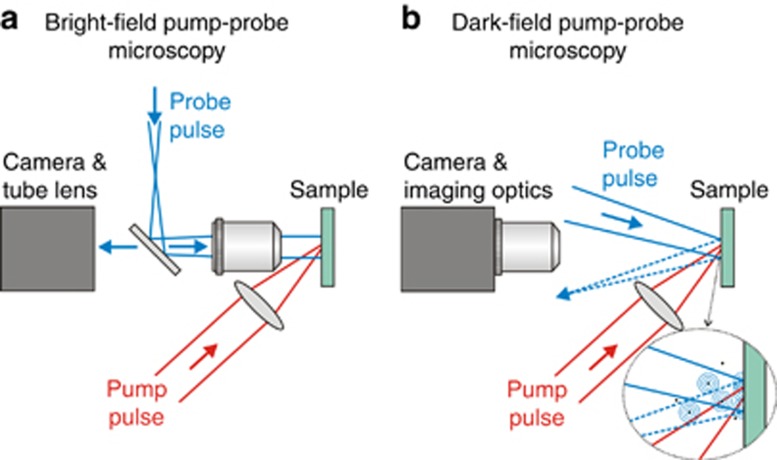
Schematics of two variants of time-resolved pump-probe microscopy for studying ultrafast laser-induced processes. A strong pump pulse (red lines) excites a material surface, which is then illuminated by a weak, delayed probe pulse (blue lines). (**a**) bright-field imaging of the sample surface using specular reflection. (**b**) dark-field imaging of optical scattering at the sample’s surface topography and by ablated particles (black dots).
